# Analytical Challenges: Determination of Tetrodotoxin in Human Urine and Plasma by LC-MS/MS

**DOI:** 10.3390/md9112291

**Published:** 2011-11-08

**Authors:** Kelvin Sze-Yin Leung, Bonnie Mei-Wah Fong, Yeuk-Ki Tsoi

**Affiliations:** 1 Department of Chemistry, Hong Kong Baptist University, Kowloon Tong, Hong Kong, China; E-Mails: fongmw@ha.org.hk (B.M.-W.F.); sct1305b@hotmail.com (Y.-K.T.); 2 Department of Pathology and Clinical Biochemistry, Queen Mary Hospital, Pokfulam Road, Hong Kong, China

**Keywords:** TTX poisoning, biological samples, ion suppression, LC-MS/MS

## Abstract

Tetrodotoxin (TTX) is a powerful sodium channel blocker found in puffer fish and some marine animals. Cases of TTX poisoning most often result from puffer fish ingestion. Diagnosis is mainly from patient’s signs and symptoms or the detection of TTX in the leftover food. If leftover food is unavailable, the determination of TTX in the patient’s urine and/or plasma is essential to confirm the diagnosis. Although various methods for the determination of TTX have been published, most of them are for food tissue samples. Dealing with human urine and blood samples is much more challenging. Unlike in food, the amount of toxin in the urine and blood of a patient is generally extremely low; therefore a very sensitive method is required to detect it. In this regard, mass spectrometry (MS) methods are the best choice. Since TTX is a very polar compound, there will be lack of retention on conventional reverse-phase columns; use of ion pair reagent or hydrophilic interaction liquid chromatography (HILIC) can help solve this problem. The problem of ion suppression is another challenge in analyzing polar compound in biological samples. This review will discuss different MS methods and their pros and cons.

## 1. Introduction

**Figure 1:**
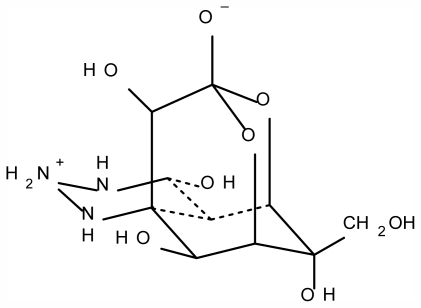
Structure of tetrodotoxin.

Tetrodotoxin (TTX), a puffer fish toxin named after the fish’s order name Tetraodontiformes, is a potent neurotoxin. It is of low molecular weight, with a unique structure (Figure 1), only relatively recently determined in 1964 [1–3]. It is a well known marine toxin, a powerful sodium channel blocker [4,5] and is found not only in puffer fish but also in some gastropods. TTX binds to and blocks sodium channel found in excitable tissues such as nerves and muscle. The inhibition of sodium entry through ion channel renders these tissues nonfunctional. TTX is about 10,000 times more lethal than cyanide by weight; the lethal dose is about 1–2 mg for adults [6,7]. There are several other marine animals, such as Gobius cringer, horseshoe crabs, Costa Rican frog of the genus Atelopus, Australian blue-ring octopus and Californian newt, all of which if eaten, can cause poisoning. TTX poisoning is frequently encountered in Japan, Taiwan and Southeast Asia including Hong Kong, where fugu or puffer fish is considered a delicacy. According to the statistics of the Japanese Ministry of Health, Labor and Welfare, during the 5 years from 2002 to 2006, 116 incidents of puffer fish poisoning occurred in Japan, involving 223 patients and 13 deaths [8]. From 1993 to 2006, 10 incidents of puffer fish poisoning were reported in Hong Kong [9] which involved 23 persons with 1 fatality. In Singapore, from 2001 to 2006, a total of 53 patients with the history of puffer fish ingestion were admitted to a regional hospital; among them, 8 died [10]. In Taiwan, from 2001 to 2004, two incidents of TTX intoxication occurred. One out of 6 patients died for the case in 2001 and 2 out of 6 patients died in 2004’s case report [11,12]. A more recent outbreak of TTX poisoning occurred in Bangladesh in 2008 which involved 141 patients, among them 17 patients died [13]. In Taiwan and China, some cases of TTX poisoning have been caused by the mistaken ingestion of the muscle tissue of a puffer fish species, by ingesting puffer roe that had been sold as fake dried mullet roe called “karasumi”, or by ingesting a dried dressed fish fillet produced from toxic puffer fish by a food processing company [14–17]. In Asia, gastropods are widely used as a traditional food, and poisoning incidents associated with nassariid gastropods have been reported occasionally in Taiwan and mainland China since 1994 [18–20]. Overall, however, the main cause of TTX intoxications reported in Japan and South-east Asia is related to puffer fish ingestion.

The causative agent of food poisoning is usually established from patient’s symptoms and identification of the toxins by analyzing leftovers of the suspected food eaten. In 1941, Fukuda and Tani provided a clinical grading system for TTX poisoning [21,22]. This four-degree classification is based on symptoms and stages of progression, and is still of clinical value (Table 1). Three factors contribute to the degree of intoxication: the amount of TTX ingested, time lag after ingestion until admission to hospital, and pre-existing diseases. First- and second-degree cases are relatively mild; there is loss of some neuromuscular and neurological function, but reflexes are still intact. Third-degree poisoning is characterized by more severe disturbances, e.g., ataxia, widespread paralysis, pronounced hyporeflexia, dropping blood pressure, cyanosis, and respiratory failure. Fourth-degree cases are severely poisoned patients presenting with cessation of respiration, decreased arterial oxygen level, unconsciousness, brandycardia, and hypotension.

Since there is no antidotes for TTX poisoning, treatment is mainly by supportive therapy and may involve mechanical ventilation for oxygen supply, normal saline infusion for distending the intravascular volume, gastric emptying procedures, or treatment with dopamine. Early diagnosis and prompt clinical management essentially contribute to low mortality rates. In Japan, the fatality rate fell from 80% at the beginning of the last century, with more than 100 deaths per year, to about 6% in the 90 s [6,23,24] due to the improvement of early life-saving systems in emergency medicine. In other Asian countries, mortality rates between 2–22% were reported [10,25–27].

Apart from diagnosing from signs and symptoms, analyzing TTX in leftover food is another way to make the diagnosis. However, leftover food is sometimes not available, either because it has not been brought with the patient to the hospital or because it has been discarded. Under such conditions, the detection of TTX in patient’s urine or blood is essential to confirm the diagnosis of TTX poisoning. As TTX is a very polar compound, the ingested toxin is mainly eliminated in urine. Previous studies have indicated that TTX only remains in the plasma component of the blood for a matter of hours (less than 24 h), but can be found in urine even on day 4 after ingestion [28,29]. In light of this, urine TTX determination could be a better choice, for the purposes of clinical laboratory diagnosis, to confirm TTX poisoning. Furthermore, in order to eliminate the effect of dehydration and variations in urinary output, creatinine adjustment should be made for all urine samples.

## 2. Challenges of TTX Determination in Human Urine and Plasma

Basically, determining TTX poisoning from body fluids faces three challenges. First, unlike in food, the amount of toxin in the urine and blood of a patient who has been poisoned is typically low; therefore a very sensitive method is required. Secondly, because TTX is a very polar compound, there will be little of any retention on conventional reverse-phase columns; thus, use of another type of analytical column such as normal phase columns or modification of mobile phase composition is required. Third, endogenous metabolites in urine are commonly very polar compounds, just like TTX, which may pose the problem of ion suppression in MS analysis. Thus, a very efficient sample clean-up procedure is required.

### 2.1. Analytical Methods for TTX Determination—A Brief Overview

The standard method accepted worldwide for determining TTX toxicity in food matrices is the mouse bioassay [30]. However, biological tests are not completely satisfactory, due to their low sensitivity and the absence of specialized variations. Moreover, there is growing resistance against the use of animals in experiments. In addition, the bioassay is not suitable for measuring TTX in a poisoned patient’s urine or blood samples. In this report we only focus on analytical methods for the determination of TTX in human urine and blood.

Several analytical methods for detecting TTX in urine and blood samples of poisoned patients have been reported, namely: gas chromatography-mass spectrometry (GC-MS) [31,32], immunoaffinity chromatography [33], high performance liquid chromatography with post-column derivatization and fluorescence detection (HPLC-FLD) [29], HPLC with ultra violet (UV) detection [34] and liquid chromatography-mass spectrometry (LC-MS) [11,35]. Apart from the chromatography based methods, TTX in biological samples can also be measured by using a specific enzyme-linked immunosorbent assay (ELISA) [36]. Table 2 summarizes several reports from Asia, but not a complete catalog of all cases of tetrodotoxication. TTX levels detected in urine and plasma/serum of patients with TTX poisoning and the analytical methods used are shown. Among these methods, the GC-MS method required a complicated extraction process and is considered time-consuming—perhaps too time-consuming when a patient’s life is at stake. HPLC with either fluorescence or UV detection is generally limited in sensitivity. The immunoaffinity chromatography method shows relatively high sensitivity in detecting TTX in urine, but requires a costly monoclonal antibody. The ELISA method uses a monoclonal antibody with high specificity for TTX with fast analysis time of about 30 min. [37], but it also requires a costly reagent. In contrast, LC-MS/MS for TTX determination in human urine and blood requires a simple sample preparation when compared with GC-MS and provides a better limit of detection when compared with HPLC-FLD or HPLC-UV methods. The running cost of LC-MS/MS is much cheaper when compared with the ELISA method. Table 3 summarizes different analytical techniques for the determination of TTX in human urine and/or blood.

### 2.2. Determination of TTX in Human Urine and Blood by ELISA

Islam *et al.* [13] measured the TTX concentrations in urine and blood from 38 patients from the large outbreaks of TTX poisoning in Bangladesh using the ELISA method according to the method of Ngy *et al.* [36] using a monoclonal anti-TTX antibody developed by Kawatsu *et al.* [37]. Samples were centrifuged at 16,100 g for 10 min, and then ultrafiltrated through an Ultracel YM-50 membrane (50,000 Da cut-off; Millpore, Bangalore, India). The filtrates were then analyzed by ELISA for TTX determination.

### 2.3. Determination of TTX in Human Urine and Blood by LC-UV

Yu *et al.* [34] developed a method of HPLC coupled with UV detection for the analysis of TTX in human urine and plasma. Samples are treated by Sep-Pak C18 cartridge (Sep-Pak cartridge Vac, Waters, Australia) followed by a weak cation exchange column. An Allsphere ODS-2, 5 μm, 250 × 4.6 mm analytical column was employed. The detection limit of their method was 10 ng/mL. Although the instrument set up is simple, the assay sensitivity may not be appropriate for patients with late admission to hospital after puffer fish ingestion.

### 2.4. Determination of TTX in Human Urine and Plasma by LC-FLD

Yasumoto and Mitishita [44] developed a method to determine TTX by HPLC with post-column derivatization and fluorescence detection in 1985. O’Leary *et al.* [29] refined their method in 2004 and used it to determine TTX in human urine and serum. Samples were cleaned up by solid phase extraction, and then analyzed by HPLC with post-column derivatization and fluorescence detection. However, post-column derivatization involves the use of hot and concentrated NaOH to convert TTX into 2-amino-6-hydroxymethyl-8-hydroxyquinazoline (so-called “C9 base”) to produce the fluorescent signal; this limits the recovery of TTX. The minimum quantifiable concentrations of TTX reported were 5 and 20 ng/mL for serum and urine, respectively. Due to the relatively poor assay sensitivity, this method is mainly used for urine specimen as urine typically contains a higher concentration of TTX than blood.

### 2.5. Determination of TTX in Human Urine and Plasma by GC-MS

TTX is soluble only in acidic alcohols and cannot be analyzed by GC/MS in its original form. TTX should be treated with alkali to form 2-amino-6-hydroxymethyl-8-hydroxyquinazoline (“C9 base”), which can then be trimethylsilylated for GC/MS analysis. For body fluid and blood plasma, additional extraction steps are required which include passing through Sep-Pak C18 cartridge twice [42]. The first Sep-Pak C18 cartridge is used only for removal of hydrophobic impurities. The second Sep-Pak C18 cartridge is used for extraction of the C9 base produced by the alkali treatment. The detection limit of this method for body fluid is 1 ng/mL and for blood plasma is 0.5 ng/mL. The lengthiness of sample preparation of this process compels researchers to look for an alternative method.

### 2.6. Determination of TTX in Human Urine and Plasma by LC-MS (MS)

This is the most popular method for determination of TTX in human urine and plasma/serum due to its superiority in assay sensitivity and ease of sample clean up. The major challenge lies in solving problems of ion suppression when analyzing TTX in urine samples by LC-MS methods. Endogenous metabolites in urine are very polar compounds, just like TTX, which may pose the problem of ion suppression in LC-MS analysis. From reports in the literature one may conclude that combined methods for sample clean up are required, either solid phase extraction (SPE) followed by ultrafiltration [11,12,39], or double SPE [38]. For the one using simple sample clean up method [43], a HILIC column was employed as analytical column for TTX separation.

Various LC-MS methods have been reported in recent years. Tsai *et al.* [11] used a Sep-Pak C18 cartridge and filtered through a 3000 MW cut-off Ultrafree microcentrifuge filter (Micron YM-3, Millipore, Waters) as sample preparation, and the detection of TTX was accomplished using positive ion electrospray ionization (ESI)-LC-MS. The analytical column was Zorax 300SB-C3, and the mobile phase was composed of 1% acetonitrile, 10 mM trimethylamine, 10 mM ammonium formate at pH 4.0. Jen *et al.* [39] used an Oasis MCX cartridge (Millipore, Waters, USA) followed by filtration through a 3000 MW cut-off ultrafree microcentrifuge filter for sample clean-up, and detection by LC-MS/MS. The study used a Waters Cosmosil Hilic column with gradient elution with 0.1% formic acid and methanol as mobile phases. The total run time was 15 min included the time for re-equilibration. The detection limit was 1 ng/mL in blood serum. Table 3 summarizes other LC-MS/MS methods.

Despite the fact that reversed phase liquid chromatography (RPLC) is overall the most used separation technique, certain solutes, especially polar and hydrophilic compounds like TTX, are not retainable in a simple fashion. Over a long period normal phase liquid chromatography (NPLC) has been the technique of choice for this purpose. The introduction of hydrophilic interaction liquid chromatography (HILIC) makes the analysis of polar compound easier. The HILIC technique bears similar features with traditional NPLC, but with the important difference that HILIC employs semi-aqueous mobile phases. Consequently, with respect to analyte solubility in the eluent and matrix compatibility, HILIC is superior, as the mobile phase compositions used are comparable to RPLC separations and thus a very MS-friendly technique. HILIC columns contain a stationary phase that is hydrophilic and often charged, at least in some region of the pH-scale. Compounds on the column interact with the stationary phase, and is generally more strongly retained than the more hydrophilic the compound. Our previous publication [38] used Superclean LC18 (Supelco, USA) and HILIC cartridges (SeQuant ZIC-HILIC SPE cartridge, Sweden) as sample clean-up procedure for the determination of TTX in human urine and plasma before detection by LC-MS/MS. We found that both Superclean LC18 and Sep-Pak cartridges could retain hydrophobic substances in the sample but could not retain TTX. The eluates from Superclean LC18 or Sep-Pak cartridges are further cleaned by the HILIC cartridge to remove interferences that eluted close to TTX peak and therefore fixed the problem of ion suppression.

Since TTX is a very polar compound, there will be lack of retention on conventional reverse-phase columns. To solve this problem, one may use an HILIC column as the analytical column as documented by Zhang and Cai; and Nakagawa *et al.* [43,45]. Our previous study [38] used an ion pair reagent, heptafluorobutyric acid, to solve the problem of analyzing a very polar compound in RPLC. An Atlantics dC18 column was used, with isocratic elution of a single mobile phase containing 10 mmol/L ammonium formate with formic acid (95:5, v/v), with 5 mM heptafluorobutyric acid and 2% acetonitrile, total run time is 5.5 min.

## 3. Correlation between Blood and Urine TTX Concentrations and Poisoning Symptoms

### 3.1. Blood TTX Concentrations and Intoxication Symptoms

Although a number of TTX intoxication case reports have been published [10,26,46,47] most do not report the urine and/or blood TTX concentrations in patients. Zimmer has tried to find correlation between blood TTX concentrations and the poisoning symptoms [48]. All available blood TTX concentrations were plotted against the intoxication grade of the corresponding patients. For those classified as first and second degree in the four-degree classification, blood levels were similar to or lower than the IC_50_ (half maximal inhibitory concentration) of TTX sodium channels. In severe cases, *i.e.*, fourth degree, TTX concentrations of 12.8–52.7 ng/mL were reported. In the recent large outbreaks of TTX poisoning in Bangladesh in 2008, 141 patients involved. Islam *et al.* [13] reported that symptoms developed after the consumption of less than approximately 50 g of puffer fish. Seven patients died after eating more than 100 g of puffer fish. They also found there appeared to be a strong correlation between blood TTX levels and the development of paralysis and respiratory arrest. Five patients who died within 15–30 minutes and 2 who died about 4 h after admission exhibited blood TTX concentrations > 9 ng/mL. Only 3 patients with TTX concentrations of 9.3–10.0 ng/mL survived. They suggested that TTX concentrations greater than 9 ng/mL are potentially lethal, leading to respiratory arrest.

### 3.2. Urine TTX Concentrations and Intoxication Symptoms

Our group previously reported a case series of 8 patients with TTX intoxication and TTX was detected in all the urine samples but not in any blood plasma [38]. This demonstrated that TTX only remains in the blood for a short time, and results agree with observations described in previous works [11,29,34]. In order to eliminate the effect of dehydration and variations in urinary output, creatinine adjustment was made for all urine samples. It was found that the creatinine-adjusted TTX concentrations (UC-TTX) in urine correlate well with the degree of poisoning. For patients with UC-TTX greater than 20 ng/μmol creatinine were found to have liver derangement as evidenced by increases in liver enzymes. A case with very high urine TTX levels (41.1 ng/μmol creatinine; 460.5 ng/mL) showed sinus rhythm with ST 1 mm elevation over V2-V4 in the electrocardiogram, suggested myocardial infarction of the anterior wall of the heart.

Although Islam’s group [13] found a strong correlation between blood TTX levels and the developed symptoms, urine TTX levels were not correlated to either blood TTX levels or intoxication symptoms. However, there was no creatinine adjustment being made for urine TTX levels, and the difference of hydration state of patients would significantly alter the excretion of TTX. The expression of absolute amount of TTX in urine alone was difficult to interpret. The findings could also be due to time differences in blood and urine sampling.

Apart from the above findings, one should bear in mind that the severity of symptoms and prognosis generally depends on the amount of toxin ingested, and the patient’s overall health and/or other medical conditions, such as diabetic neuropathy, neural myoinositol and Na-K-adenosine-triphosphatase deficiency [49,50] as well as natural variation in individual sensitivity to the toxin.

## 4. Conclusions

The application of LC-MS/MS to clinical analysis has evolved considerably since it was first used, both in research facilities and for routine analysis in clinical biochemistry laboratories. Initially GC-MS was used for biological analysis, but the requirement that GC needs a volatile analyte meant that elaborate extraction and derivatization protocols were needed for analysis of the typically polar, involatile biomolecules found in clinical samples. LC is a much more appropriate separation technique for these polar molecules found in biological samples. It also reduces the need for derivatization to volatile molecules. It is the coupling of LC to MS that led to the adoption of clinical MS. However, the determination of small polar molecules in biological samples is often challenging due to the lack of retention on conventional reverse-phase columns and significant ion suppression due to endogenous substances in human urine and plasma. The development of the HILIC stationary phase, as an analytical column or in sample preparation cartridge, partly helps solve this problem. With continuous improvement and advancement in LC and MS technology, it is expected that determination of other toxins in human urine and blood plasma will soon be published.

## Figures and Tables

**Table 1:** Clinical grading system in TTX intoxication according to [21,22].

Degree	Signs and Symptoms	Onset
First	Perioral numbness and paraesthesia, with or without gastrointestinal symptoms (mainly nausea).	5–45 min
Second	Lingual numbness, numbness of face, and other areas (distal). Early motor paralysis and in-coordination. Slurred speech. Normal reflexes.	10–60 min
Third	Generalized flaccid paralysis, respiratory failure, aphonia, and fixed or dilated pupils. Patient is conscious.	15 min– several hours
Fourth	Severe respiratory failure and hypoxia. Hypotension, bradycardia, and cardiac dysrhythmias. Unconsciousness may occur.	15 min–24 h

**Table 2:** Comparison of analytical methods used in detecting TTX levels in Asian patients, 1989–2008.

Incident Time	Location	Patients Involved	Detection Method	LOD (ng/mL)	TTX Conc. in Urine (ng/mL)	TTX Conc. in Plasma/Serum (ng/mL)	Reference
2008	Bangladesh	141	ELISA	Not mentioned	0.4 to 75.4	<1.6 to 13.7	[13]
2007	Hong Kong	1	LC-MS/MS	0.13	88.2	N/A	[38]
2006	Hong Kong	4	LC-MS/MS	0.13	30.7 to 460.5	<0.13	[38]
2006	Taiwan	3	LC-MS/MS	0.1	N/A	3.3	[39]
2005	Hong Kong	3	LC-MS/MS	0.13	59.3 to 109.6	< 0.13	[38]
2005	Taiwan	6	LC-MS	1.0	169 to 325	<1 to 8	[12]
2004	Japan	7	LC-MS/MS	0.1	15 to 150	0.9 to 1.8	[40]
2001	Taiwan	6	LC-MS	4.9	15 to 109.7	<4.9 to 13	[11]
1989–1996	Japan	6	HPLC-FLD	2.0	6 to 102	N/A	[33]
Not mentioned	Japan	11	GC-MS	0.5	15 to 650	2.5 to 320	[41]

N/A: not available.

**Table 3:** Summary of different analytical techniques for TTX determination in human urine and/or blood.

Analytical Method	Analytical Column	LC Mobile Phase	Sample Preparation		LOD (ng/mL)	Reference
			Urine	Plasma/Serum		
ELISA	-	-	Ultrafiltered through an Ultracel YM-50 membrane (50000 Da cut-off)	Same as urine	Not mentioned	[13]
LC-UV	Allsphere ODS-2, 5 μm, 250 × 4.6 mm	1-heptanesulfonic acid sodium salt monohydrate, sodium dihydrogen phosphate anhydrous, methanol	SPE: C18 Sep-Pak and HiTrap (weak cation exchange) columns	Same as urine	10	[34]
LC-FLD	Nova-Pak C18, 4 μm, 100 × 8 mm	Heptanesulfonic acid, acetonitrile	SPE: C18 Sep-Pak and Strata X-C cation Mixed-mode polymer cartridge	Oasis MCX	s: 5; u: 20	[29]
LC-FLD	Inertsil ODS-2, 250 × 4.6 mm	Sodium potassium phosphate with sodium dodecyl sulfate	SPE: Bond Elut SCX cartridge	N/A	Not mentioned	[42]
GC-MS	DB-5 fused silica capillary column, 30 m × 0.25 mm i.d. film thickness 5 μm	-	Sep-Pak C18 columns	Similar to urine	s: 0.5; u: 1	[42]
LC-MS/MS	Atlantics dC18, 5 μm, 150 × 2.1 mm	Heptafluorobutyric acid, ammonium formate with formic acid, acetonitrile	SPE: Sep-Pak C18 and HILIC columns	Same as urine	0.13	[38]
LC-MS	Zorax 300SB-C3, 150 × 4.6 mm	Trimethylamine, ammonium formate, acetonitrile	SPE: Sep-Pak C18 and ultrafiltration	Same as urine	4.97	[11]
LC-MS/MS	Cosmosil Hilic 150 × 4.6 mm	Formic acid, methanol	N/A	SPE: Oasis MCX and ultrafiltration	0.1	[39]
LC-MS/MS	GC Science ODS-3 3 μm, 50 × 2.1 mm	ion pair reagent and methanol	Methacrylate-styrenedivinylbenzene cartridge	Same as urine	0.1	[40]
LC-MS/MS	Zorbax 300SB-C3, 150 × 4.6 mm	Trimethylamine, ammonium formate, acetonitrile	Sep-Pak C18 and ultrafiltration	Same as urine	1	[12]
LC-MS/MS	TSK-gel amide-80	Ammonium formate, methanol, acetonitrile	Protein precipitation	Same as urine	s: 0.3; u: 1	[43]

LOD: limit of detection; SPE: solid phase extraction; N/A: not applicable; s: serum; u: urine.
